# Spontaneous regression of a chiari malformation type 1 in a 58-year-old female

**DOI:** 10.1259/bjrcr.20160016

**Published:** 2016-07-28

**Authors:** Trevor Gaunt, Sharief Aboelmagd, Hilmar Spohr, Janak Saada

**Affiliations:** Norwich Radiology Academy, Norfolk and Norwich University Hospitals NHS Foundation Trust, Norwich, UK

## Abstract

Studies have established a complex age-related variation of the position of the cerebellar tonsils relative to the foramen magnum (FM). Chiari malformation type 1 (CM1) is generally defined by a protrusion >5 mm from the FM and may be an asymptomatic incidental finding. Symptoms include headache, nausea and neurological disturbances, including trigeminal neuralgia. Moreover, tonsils are often peg shaped and associated with syringohydromyelia. Symptomatic CM1 may be managed with decompression of the posterior cranial fossa, but spontaneous regression in adults has been reported occasionally. Theories include restoration of normal cerebrospinal fluid dynamics around the FM after rupture of subarachnoid adhesions or the syrinx itself during transient episodes of raised intracranial pressure. Supratentorial neurosurgery has also been implicated. We present a 58-year-old female diagnosed with CM1 and no associated syringohydromyelia following MRI investigation of trigeminal neuralgia. Managed medically, she re-presented 6 years later with new neurological symptoms. A subsequent MR study of the posterior cranial fossa showed resolution of the CM1, with only residual tonsillar ectopia. At no point was intracranial intervention performed, nor were there any events that might favour CM1 regression. This case demonstrates spontaneous resolution of CM1 without surgical intervention.

## Background

According to the translation by Radkowski,^1^ Hans Chiari described a group of pathologically different congenital hindbrain abnormalities in 1891. The most common malformation, Chiari malformation type 1 (CM1), is described as the downward displacement of peg-like cerebellar tonsils^2^ below the foramen magnum (FM) into the upper cervical canal. It has been associated with a volumetrically small posterior cranial fossa,^3^ distortion of the bulbomedullary junction and syringohydromyelia (SHM). Studies have established a complex age-related variation of the position of the cerebellar tonsils relative to the FM.^2^ The relative position of the inferior margin of the cerebellar tonsils to the FM is subject to change with differential growth of the brain tissue and the developing skeleton. The diagnostic features on imaging of CM1 are debated but the most frequently quoted criteria include a peg-like rather than a rounded morphology of the cerebellar tonsils^2^ with >5 mm descent below the FM.^4,5^

Clinical presentation of CM1 varies from an asymptomatic incidental finding^6^ to headaches, visual disturbance, dizziness, tinnitus, dysphagia and other cranial neuropathies, including trigeminal neuralgia.^3^ CM1 is often associated with SHM.^7–16^ When symptomatic, it can be managed by a range of neurosurgical procedures, including posterior fossa decompression, with the aim of restoration of normal cerebrospinal fluid (CSF) flow dynamics in the region of the FM.

Spontaneous regression has been reported in children,^16–20^ and is likely due to posterior cranial fossa expansion with normal cranial growth.^9^ Cases of spontaneous CM1 regression in adults has seldom been reported.^7–10,21^ Theories postulate the relief of raised intracranial pressure by restoration of normal CSF flow around the FM. This may follow rupture of subarachnoid adhesions^7^ or rupture of the syrinx into the subarachnoid space^22^ in the context of childbirth, or after supratentorial neurosurgery.^10^

We report a unique patient with trigeminal neuralgia and cerebellar tonsils positioned 25 mm below the FM, which regressed spontaneously without neurosurgical intervention.

## Case report

### Presentation

In September 2013, a 58-year-old female presented to her general practitioner with progressive paraesthesia in her lower limbs. Her past medical history included asthma, well-controlled Type 2 diabetes, and a 6-year history of trigeminal neuralgia and CM1.

**Figure 1.**
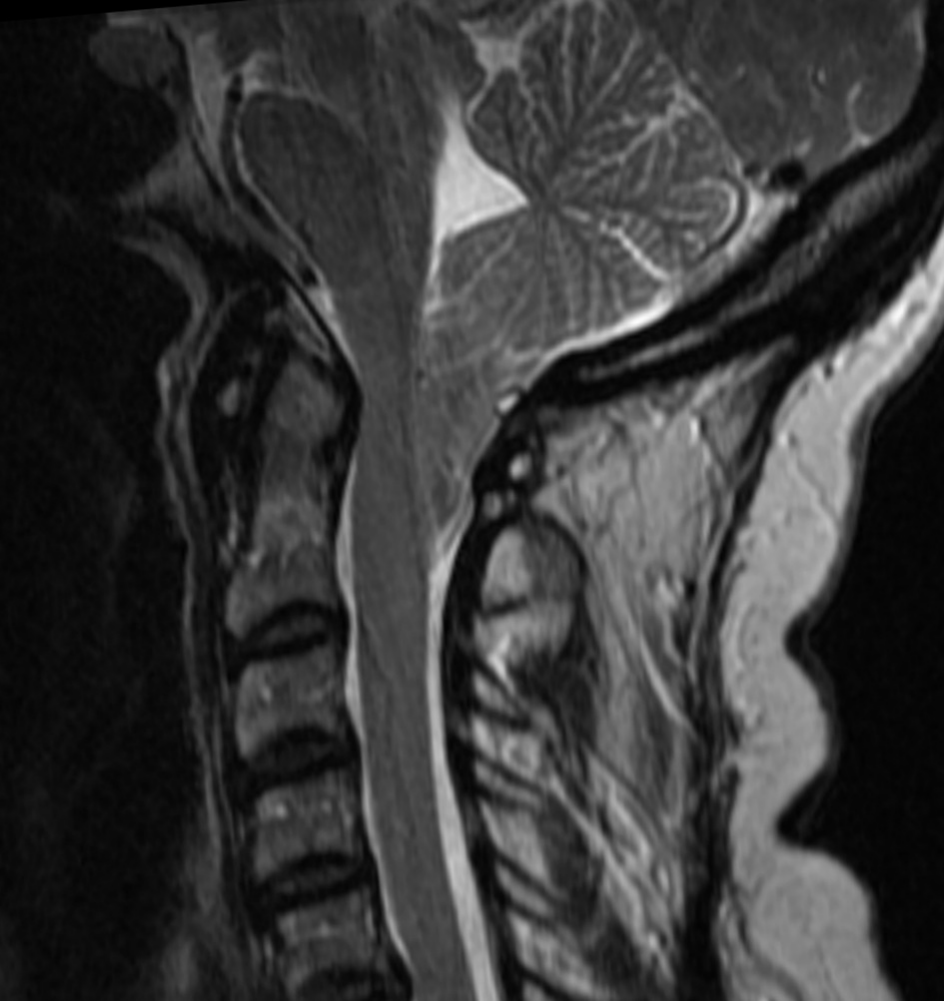
Sagittal section *T*_2_ weighted MR image of the upper cervical spine and posterior cranial fossa demonstrating peg-like cerebellar tonsils positioned 25 mm beyond the foramen magnum.

A cranial MRI in June 2007 had shown no neurovascular conflict or trigeminal nerve disruption. However, the cerebellar tonsils were positioned 25 mm below the FM, consistent with CM1, in addition to compression of the medulla oblongata and flattening of the anterior surface of the pons. The posterior fossa was mildly underdeveloped but there was no evidence of SHM (Figure 1).

### Investigations and diagnosis

**Figure 2.**
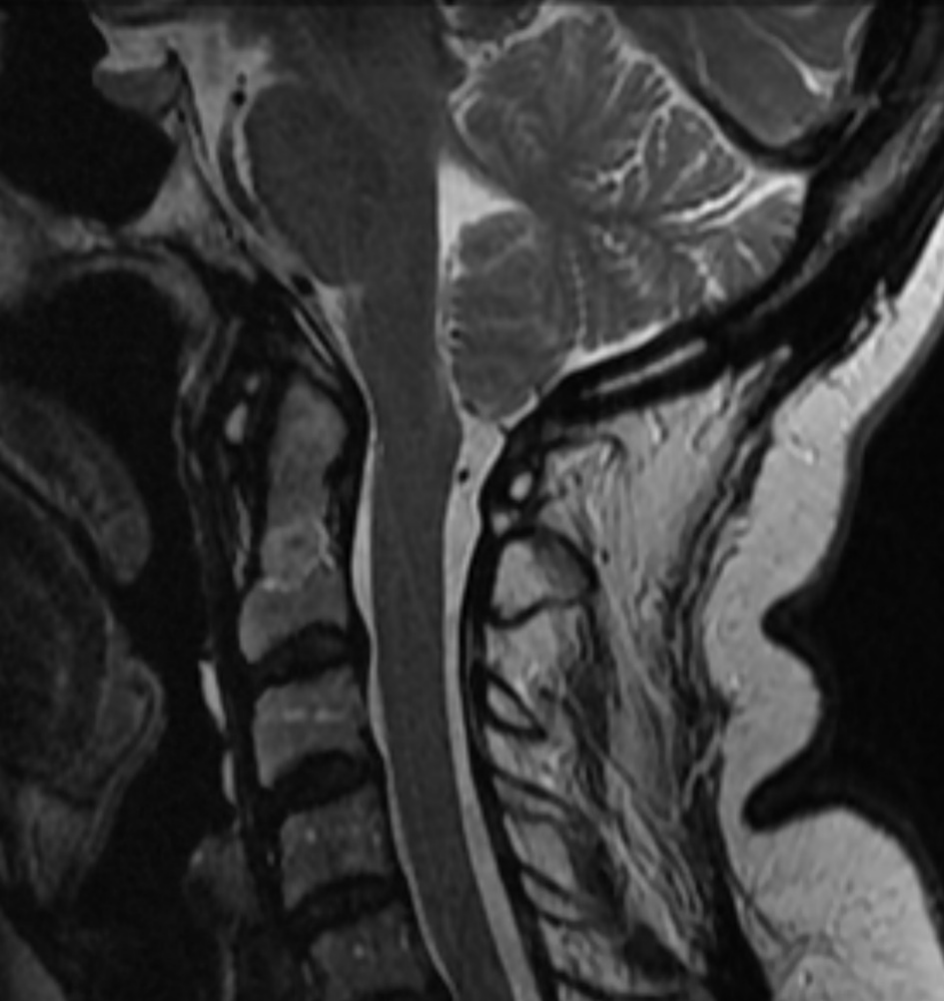
Sagittal section *T*_2_ weighted MR image of the upper cervical spine and posterior cranial fossa demonstrating normal tonsillar position and morphology.

At presentation in 2013, her facial pain was medically controlled with carbamazepine (400 mg twice daily) but could be triggered with a pinprick in the left maxillary region. The remainder of her neurological examination and nerve conduction studies were normal. Surprisingly, a follow-up MRI showed that the CM1 had resolved (Figure 2).

### Treatment and outcome

The patient’s lower limb paraesthesia resolved spontaneously within a matter of weeks, with no identifiable cause. By August 2015, her trigeminal neuralgia was once again well controlled with carbamazepine (400 mg twice daily), with no episodes of breakthrough pain. At no point was surgery or any other intervention performed.

## Discussion

Tonsillar position beyond the FM in the sagittal plane can be described using McRae’s line^23^ drawn between the basion and opisthion on a sagittal *T*_2_ weighted MR image.

Our patient demonstrated dramatic spontaneous regression of CM1 on serial imaging. Regression of CM1 has been reported in children^16–20^ and the cause is likely to be normal expansion of the posterior cranial fossa with growth.^9^

We have excluded the possibility that the low-lying tonsils at initial presentation were due to undiagnosed spontaneous intracranial hypotension. Our patient lacked any appropriate previously described clinical or radiological features such as new positional headaches, effacement of the suprasellar cisterns, downward displacement of the brain or subdural fluid collections.^20,24^

Of the comparable five cases reported in adults,^7–10,21^ SHM of the spinal cord was present in four^7–10^ and improved with regression of CM1. Each case reported the presence of neurological symptoms. Trigeminal neuralgia was present in cases reported by Klekamp et al^7^ and Coppa et al,^9^ diplopia on left-sided gaze was reported by Miele et al^10^ and ataxia and dizziness were reported by Briganti et al.^21^ In each case, the patient was asymptomatic at follow-up. These differ from our case in that our patient’s symptoms have persisted, but remain controlled with carbamazepine.

In some cases, CM1 regression has followed certain predisposing factors.^21^ Miele et al^10^ reported the regression of a CM1 following supratentorial craniotomy for the excision of cavernous malformation, which by nature of the operation may favour a pressure-related tonsillar regression. Similarly, Coppa et al^9^ reported a case following an episode of CSF otorrhoea. Muthukumar and Christopher^8^ reported a case following normal vaginal delivery. This may be accounted for by intracranial and intraspinal compartment pressure changes during labour^5,10^ with subsequent rupture of subarachnoid adhesions. Other suggested causes for spontaneous regression include the varying position of cerebellar tonsils with age^25^ and cerebellar atrophy.^12^

Klekamp et al^7^ reported the complete regression of a CM1, with no predisposing factors; however, this was associated with the resolution of a concomitant syringomyelia. Briganti et al^21^ reported a case in a 62-year-old female with no predisposing factors and without an SHM, and is the only case similar to ours. However, the patient in question had symptoms of dizziness and ataxia at presentation rather than trigeminal neuralgia.

Our case is unique in that there were no factors that may have favoured regression; she had no concomitant SHM and her trigeminal neuralgia persisted. Aside from the patient reported by Briganti et al,^21^ other patients were under 40 years of age,^7–10^ whereas our patient was in her late 50s. The time interval reported between diagnosis and regression is between 32 and 48 months. In our patient’s case, the time interval was 6 years. This is probably due in part to well-controlled pain and her re-presentation with new, albeit transient, neurological symptoms. It is likely that without this presentation, she would not have undergone any additional imaging.

The optimal management for CM1 is still unclear. It has been recommended that patients demonstrating spontaneous regression of CM1 should be followed up owing to the possibility of recurrence.^11,21^ Asymptomatic CM1 is managed conservatively, and symptomatic patients with posterior fossa decompression if medical management fails. The frequency of spontaneous regression is low, but in light of our case, it may be prudent to check if CM1 is still present should intervention be delayed.

## Conclusions

We have presented a rare case of CM1 regression, which differs from those in the current literature. Other cases are associated with resolution of neurological symptoms, SHM or predisposing factors. It further demonstrates spontaneous resolution of CM1 without the need for surgical intervention. The mechanism is intriguing and not well understood.

## Learning Points

1. CM1 is defined by cerebellar tonsil position >5 mm from the FM.
2. It is commonly asymptomatic but may be associated with neurological symptoms such as pressure headache, dizziness and trigeminal neuralgia.
3. It is often associated with SHM.
4. Treatment is conservative or *via* decompression of the posterior cranial fossa.
5. Spontaneous regression of CM1 can occur rarely.

## Consent

Written informed consent for the case to be published (including images, case history and data) was obtained from the patient for publication of this case report.
